# Human-centred cyber secure software engineering

**DOI:** 10.1007/s41449-022-00346-2

**Published:** 2022-12-23

**Authors:** Karen Renaud

**Affiliations:** 1 University of Strathclyde, Livingstone Tower, 26 Richmond Street, G1 1XH Glasgow, UK; 2 Rhodes University, Grahamstown, South Africa; 3 University of South Africa, Pretoria, South Africa; 4 Abertay University, Dundee, UK

**Keywords:** Software Engineering, Cybersecurity, Human Factors, Softwareentwicklung, Cybersicherheit, Menschliche Faktoren

## Abstract

Software runs our modern day lives: our shopping, our transport and our medical devices. Hence, no citizen can escape the consequences of poor software engineering. A closely-aligned concern, which also touches every aspect of our lives, is cyber security. Software has to be developed with cybersecurity threats in mind, in order to design resistance and resilience into the software, given that they are often rooted in malicious human behaviour. Both software engineering and cyber security disciplines need to acknowledge and accommodate humans, not expect perfect performances. This is a position paper, delineating the extent of the challenge posed by this reality, and suggesting ways for accommodating the influence of human nature on secure software engineering.

*Practical Relevance*: Socio-technical systems are made up of people, processes and technology. All can fail or be suboptimal. Software itself, being designed, developed and used by humans, is likely to malfunction. This could be caused by human error, or by malice. This paper highlights this reality, taking a closer look at all of the possible sources of malfunctioning technology. By doing so, I hope to infuse the management of socio-technical systems with an understanding and acknowledgement of this reality.

## Abstract (de)

Software steuert unser modernes Leben: unsere Einkäufe, unsere Transportmittel und unsere medizinischen Geräte. Daher kann sich kein Bürger den Folgen schlechter Softwareentwicklung entziehen. Ein eng damit verbundenes Anliegen, das auch jeden Aspekt unseres Lebens berührt, ist die Cybersicherheit, und Software muss unter Berücksichtigung von Cybersicherheitsbedrohungen entwickelt werden, um Widerstandsfähigkeit in die Software zu integrieren. Sowohl Software-Engineering als auch Cybersicherheitsdisziplinen sind für die Verwendung durch Menschen konzipiert, nicht für unfehlbare Roboter. Dies ist ein Positionspapier, das das Ausmaß der Herausforderung beschreibt, die sich aus dieser Notwendigkeit ergibt, und Wege aufzeigt, wie dem Einfluss der menschlichen Natur auf cybersicheres Software-Engineering Rechnung getragen werden kann.

*Praktische Relevanz*: Soziotechnische Systeme bestehen aus Menschen, Prozessen und Technologie. Alle können fehlschlagen oder suboptimal sein. Software selbst, die von Menschen entworfen, entwickelt und verwendet wird, weist wahrscheinlich Fehlfunktionen auf. Dies kann durch menschliches Versagen oder durch Vorsatz verursacht werden. Dieses Papier beleuchtet diese Realität, indem es alle möglichen Ursachen für fehlerhafte Technologie genauer unter die Lupe nimmt. Auf diese Weise hoffe ich, das Management soziotechnischer Systeme mit einem Verständnis und einer Anerkennung dieser Realität zu erfüllen.

## Introduction

Consider that every aspect of our lives is influenced by invisibly functioning software. For example, when we shop, software runs the tills; it ensures that the temperature remains constant, and so-called “smart” devices track our movement through the store to identify the most effective product placement strategies. Our hospitals use Internet-connected devices to carry out tests, and our airplanes and cars have embedded computers. Underlying all of this is software, and such software is developed by software engineers.

Software engineering is a challenging, complex and error-prone activity. Sometimes, flawed and/or insecure software is produced (Clark 2021; Collins 2009). This has been the case ever since the first software code was written (Martin 2022). Why does this happen? IBM Software Engineer Rupert Brooks wrote a book titled: “*The Mythical Man Month*” to help those working in software engineering to understand the human problems of this craft. He was one of the first software engineers to draw attention to the importance of human limitations and management and the consequences of ignoring these.

When software has errors or vulnerabilities, it can lead to widespread harms—and can even lead to loss of life. The widespread 2017 WannaCry attack brought many of the UK’s National Health System board to a near standstill, diverting funds to recovering from the attack, delaying surgeries and other treatments. The 2021 Pipeline attack disrupted the flow of critical petroleum products across the East Coast of America^1^, and led to increased gasoline prices. The consequences of these attacks are non-trivial.

This paper seeks to explore the extent of the problem, and to provide suggestions for ameliorating the impact of human fallibility on the software engineering process. The following research questions will be addressed:

### RQ1

How does human fallibility impact the secure software development process?

### RQ2

How can the impact of human fallibility be ameliorated during the secure software engineering process?

The next section will first introduce the core concepts of “software engineering” and “cyber security”. Sect. 3 will review related research about secure software engineering. Sect. 4 then addresses RQ1, Sect. 5 considers RQ2, and Sect. 6 concludes.

## Definitions

Software engineering is defined by Humphrey (1988) as “*the disciplined application of engineering, scientific, and mathematical principles and methods to the economical production of quality software*” (p. 82). The definition makes it clear that the process is challenging, with many opportunities for errors to introduce bugs into the software system.

Zhang et al. (2005) points to the need for a focus on usefulness, utility, and usability during the software engineering process. *Usefulness* of a computer system is related to the extent to which it assists users in a achieving their desired goals (Nielsen 1993). To deliver usefulness, the system has to have the correct functionality built into it (utility) (Grudin 1992) which allows users to achieve their goals with effectiveness, efficiency and satisfaction (usability) (ISO, in Bevan 2001).

*Secure* software engineering is even more challenging, given that the developer has to anticipate attack vectors, and foil the efforts of a globally distributed and innovative cyber criminals. Even if the software has been rigorously tested and is error free and usable, that does not mean that it cannot be compromised by cyber criminals in the future. Previously unknown so-called “zero-day” vulnerabilities are continuously discovered and exploited by hackers. If software starts to behave erratically, it could be due to errors but also reflect an intrusion by cyber criminals exploiting a vulnerability (Peisert et al. 2021; Zetter 2014). The widespread 2017 WannaCry and Stuxnet attacks exploited this kind of newly-discovered vulnerability (Mohurle and Patil 2017; Langner 2011).

Storey et al. (2020) argue that there is a need for research that aims to understand the human and social aspects of software development practice. Without this understanding, and targeted interventions that build on this, flawed software systems will result, systems will fail, and cyber criminals will compromise software systems.

## Related research

To consider the current state of play, a search of the research literature was carried out, using the keyword: *secure and “software engineering”.* Papers returned by Google Scholar and Scopus were filtered to ensure that only those addressing the research question were retained for analysis (66 papers). Thematic analysis was used to extract themes and to group papers into these themes. The following theses emerged:

1. Software Design and Implementation:
  - The need for better techniques for formulating desirable security properties (Devanbu and Stubblebine 2000). This might include the use of security patterns (Van Niekerk and Futcher 2015).
  - Developers needing to be trained to engage in secure software engineering and to detect vulnerabilities in their software (Braz et al. 2021; Essafi et al. 2006; Walden and Shumba 2006; Jayalath et al. 2020; Kanniah and Mahrin 2016; Arora et al. 2021; Stamat and Humphries 2009; Hein and Saiedian 2009).
  - The need for security to be part of the software engineering process from design through to implementation is highlighted (Mouratidis et al. 2005; Mellado et al. 2007; McGraw 2004; Devanbu and Stubblebine 2000; Moyón et al. 2020; Kreitz 2019).
  - The need for empirical research in secure software engineering (Cruzes and ben Othmane 2017).
2. Software Deployment
  - The need to pay attention to secure configuration of software systems (Sayagh et al. 2018), which the authors contend does not receive as much attention as it should.
3. Software Maintenance and Evolution:
  - A particular challenge is long-term maintenance of software systems, which becomes difficult because of “*imprecise, incomplete and arbitrary documentation*” [p. 320] (Villarroel et al. 2005). Devanbu and Stubblebine (2000) calls for the development of “automated, robust, flexible infra-structures for post-deployment system administration” [p. 225].
  - The need to ensure that systems remain secure as they evolve over time (Felderer et al. 2014).
4. Mentioning user issues:
  - *Software Engineers*:
    i. The cognitive demand on software engineers developing secure software is overwhelming (Apvrille and Pourzandi 2005; Giorgini et al. 2005; Mellado et al. 2007; Miller et al. 2013).
    ii. Most research focuses on the technical aspects of software engineering, neglecting human factors (Storey et al. 2020) e.g., Khan et al. (2022).
    iii. Importance of collaboration and team dynamics highlighted: (Arora et al. 2021), and of the need for communication between stakeholders during the development process (Kanniah and Mahrin 2016).
  - *End Users*: Need to consider the impact of security measures on end users (Flechais et al. 2003).

It is clear from this review that not much attention has been paid to the impact of the human factor in secure software engineering beyond pointing out that the entire process is challenging. Certainly, there is no explicit review on the impact of the human factor in the software engineering process.

We now proceed to address the two research questions.

## How our humanity influences secure software engineering

Whenever humans are involved in any process, errors are certain to occur (Reason 1990; Adams and Sasse 1999) and sometimes bad actors will behave maliciously and compromise the system’s functioning (De Cremer 2009). The established fields of medicine and civil engineering (± 1960) have long acknowledged this reality (Berry 2022; Gawande 2010; Xie and Qu 2018; Whittle and Ritchie 2000). Human tendencies to make mistakes and to act maliciously can also compromise the software engineering process. Yet, for most of the history of software engineering, this human “factor” has been neglected (Sutcliffe 1997; Storey et al. 2020).

This section addresses the first research question: *How do human factors impact the secure software development process?*

To answer this question, we now consider the full range of different stakeholders (developer, managers, deployers, end users), and the impact of human factors in each different case.

### Software developers

The software developer is at the core of the software engineering process, and the impact of their humanity has not received very much attention from researchers. To frame our discussion, we considered two lenses: personality and human needs.

In considering the first lens, we discovered that a number of studies had investigated the types of personalities of people who go into a career of software engineering. However, Cruz et al. (2015) found that “*the evidence is weak and in many cases inconclusive*” [p. 108]. Hence, instead of looking at personality, let us consider how a software engineering career satisfies the human needs of software developers: mastery, autonomy and relatedness (Ajzen 1991), and how their humanity influences the satisfaction of these. It is worth noting that the same approach could be used for the other stakeholders. However, the primary focus here is on software developers.

#### Mastery/Competence

Software engineering is a career that requires continuous learning, is creative and immensely satisfying when the project has been completed and the product prepared for deployment. Software developers, as keen problem solvers, enjoy putting pieces of code together to produce a working piece of software (Wynekoop and Walz 2000; Groeneveld et al. 2020). Brooks (1975) says: “*The programmer, like the poet, works only slightly removed from pure thought-stuff”* (p. 7).

Russo et al. (2022) find that software engineers have a higher need for cognition than the general public. The software engineer might be the adult who used to love to build lego as a child (Bialski 2017). Certainly, Stoilescu and Egodawatte (2010) explain that successful professionals enjoying “playing” with computers. As such, the software developer career is almost custom built to satisfy these individuals’ needs for mastery.

Yet, there are also challenges. Brooks (1975) explains that the need for perfect performance is one of the most difficult for humans to adapt to. Computer code does not tolerate any imperfections, so programmers have to get used to going over their code repeatedly in order to arrive at that level of perfection which is required.

Errors in software code are colloquially referred to as “bugs”. This term was coined by Grace Hopper, a United States Navy rear admiral. She found an insect (termed a bug) in a computer, which was interfering with its functioning. She promptly pasted it into the log book, where it remains for posterity. Ever since, programmers have referred to software errors as ‘bugs’ and their elimination as ‘debugging’. Brian Kernigan^2^, one of the early giants of the software engineering field, said: “*Debugging is twice as hard as writing the code in the first place*”. Debugging is the price programmers play for experiencing the pleasure of the creative programming process. Programmers who possess the ability to persevere and detect these bugs (Stolee et al. 2011) will achieve the level of mastery that is required, and are likely to remain in software engineering.

The testing phase is undertaken by others, and is trying for the programmer. He/she has spent countless hours developing the software and debugging it. Such personal investment makes him/her feel a sense of ownership: their code is “their” thought-child (as described by Brooks). That being so, it is hard for them not to feel defensive when a tester uncovers errors. It points to the programmer’s failure and can be perceived as an attack. Often, programmers are under tremendous pressure from their managers to produce the software to unrealistic deadlines. In these cases, testing may be seen as a luxury. If it is done, many errors are likely to be uncovered. This is an unwanted outcome for any software developer.

The (recent) mandate to code securely challenges their sense of mastery. Arvind Krishna, CEO of IBM^3^ said that: “*Cybersecurity is the issue of the decade. I think that is the single biggest issue we all are going to face*”. While he was talking about the role of the chief information officer, this applies equally to the software engineer. The need to develop secure software is relatively recent and many who have been in the industry for some years will not have been trained to do this.

Even if software developers master the complex software development process, the need to develop *secure* software might well not come as easily. Developing secure software requires developers to anticipate vulnerabilities, which requires something of a different mindset (Harvey et al. 2016). Anu et al. (2020) suggest that in many cases vulnerabilities that are exploited by hackers can be traced to the same handful of programmer errors. It might be that traditional software engineering training has not yet incorporated sufficient training in preventing vulnerabilities, as highlighted in the previous section. In this case, their perceived mastery is illusory—something Johari calls an “unknown, unknown” (Shenton 2007).

In relatively rare cases, software engineers might behave maliciously, for a variety of reasons (Warkentin and Willison 2009). They might insert so-called “malware” into software, which can be exploited at will (Wu 2020). On the other hand, sometimes software developers can be pressured by their managers into inserting “back doors” to ease subsequent access (Osterweil 2016). If they have not had ethical training, or feel unable to resist for fear of losing their jobs, the resulting software is inherently insecure and will permit intrusions.

Software is routinely tested to ensure that it delivers the required functionality, and is usable by its target user group. It is also often tested for known top security risk vulnerabilities^4^, so-called *penetration testing*. However, an oft-neglected kind of testing is to reveal deliberately-introduced malware (Agrawal et al. 2010).

Hence, software engineers seem to satisfy their mastery needs in their careers, but sometimes find such satisfaction frustrated by the intensely demanding expectation of near perfection in their discipline, and the need to code defensively to prevent breaches by cyber criminals.

#### Autonomy

With respect to autonomy, it is harder for software engineers to satisfy this need. The programmer does not set his/her own objectives, goals or the technologies and tools to be used. The creativity they so revel in is actually severely constrained. Programmers are similar to actors and actresses—they are the visible agents, but others set the scene and finely choreograph their actions. Brooks (1975) points out how painful this dependence is for the creative programmer, driving towards mastery of their field, especially when requirements are poorly defined or unrealistic.

The other challenge to deal with is continuously changing requirements. Such changes are unpleasant and not eagerly anticipated (Harker et al. 1993). The software engineer has to learn to tolerate uncertainty, and to not have their need for autonomy satisfied (Kalliamvakou et al. 2017). This is difficult for most humans, and many people become burnt out as a consequence of long-term uncertainty (Kuhn et al. 2009). Such burn out can lead to widespread negative outcomes for the software developer and organisation (Nesher Shoshan and Sonnentag 2020).

#### Relatedness

This term is defined by Merriam-Webster as “*having close harmonic connection—used of tones, chords, or tonalities*”. In essence, people need to spend quality time with other people—especially their loved ones and friends (McLeod 2007). Yet, the software engineering career is characterised by overwork and burnout (Afzal 2016). This will prevent them from spending time with others and from being able to relax when they do.

There is a myth that suggests that software developers are anti-social and uncommunicative—and this argument could be used to argue that it is acceptable for software developers to work excessive hours alone staring at a screen. That this is indeed a myth is highlighted by Chattopadhyay et al. (2021), who found that developers would use vlogs to challenge the misconceptions and stereotypes around their identities. Indeed, as Rodeghero et al. (2021) show, developers have a keen sense of their need to be socially connected to their colleagues and programmers with greater openness perform better in their jobs (Salleh et al. 2014; Rehman et al. 2012). If they do not get this, they will experience feelings of vulnerability (Hawkley and Cacioppo 2010), which will impact social cohesion and information sharing between employees (Searle and Renaud 2023).

#### Summary

It is clear that software developers’ jobs are unlikely to satisfy their human needs. In this situation, they will not perform optimally and if they become burnt out many negative consequences will occur, which will affect their ability to produce bug free and secure code. Managers can alleviate the issues mentioned in this Section if they are aware of them.

### Managers

Inadequate management and a lack of resources can be the cause of software development failures (Oz 1994; Aeon et al. 2021; Linberg 1999). It is also the case that many of those who manage software engineering teams do not have much technical expertise themselves (Dzuiba 2010; Kalliamvakou et al. 2017). Hence, they fail to understand the complexity of the software development process and produce unrealistic schedules (Linberg 1999).

Kalliamvakou et al. (2017) provides an extensive list of attributes of great managers of software developers. These include building a team culture, fostering communication and being available to team members. In terms of effective management, Wang and Lai (2001) point to the crucial nature of requirements management, one of the key responsibilities of managers. Wang and Lai explain that whereas changing requirements are a fact of life, the changes have to be *managed* (Bhatti et al. 2010), not merely passed down to developers.

Assal and Chiasson (2019) find that security-related software development issues often stem from a lack of organizational or process support in terms of incorporating security info software development tasks. This is something managers ought to provide, but it seems that they do not do this.

Given the need for software developers to know how to develop secure software, it is also the responsibility of the manager to ensure that they get the necessary training to help them to develop these skills. Making them aware of their lack of knowledge in this area is a clear management responsibility, because an appreciation of the lack of expertise in this area is the first step towards a willingness to having their code tested to uncover vulnerabilities (Howard and Lipner 2006). This allows “*unknown unknown*” to become a “*known unknown*”.

Afzal (2016) argues that many developers do not leave their jobs, but rather leave bad managers. This points to the crucial role of management in the software engineering process.

### Deployers

Given the opportunity for error during every stage of the software development process, there is a very high likelihood of hidden bugs remaining in software after deployment. Those who deploy software should have a rigorous maintenance process in place. As users report issues, these can then be addressed.

However, some errors are intermittent and hard to prove. When software produces anomalous outcomes, it is often the case that the first reaction is denial or to blame the operator (Clark 2021). Indeed, the latest manifestation of this kind of denial occurred in the Post Office case in the UK (Renaud et al. 2021a). Over the course of two decades, the Horizon software rolled out by the UK’s Post Office generated phantom transactions, and the Post Office unquestionably jumped to the conclusion that reported shortfalls were due to fraud (Wallis 2021). Over 700 innocent post masters were prosecuted, some were incarcerated, and many lost their livelihoods. The Post Office steadfastly refused to consider the fact that their software might be malfunctioning, which was indeed the case.

There is often naïvety amongst those who deploy software without understanding the mechanisms of software engineering. Many believe that software always works correctly (Crown Prosecution Service 2017; UCL Laws 2021), perhaps because the alternative is unpalatable and would complicate their lives and jobs. Even so, the human factor influencing the development of software should be acknowledged so that a measure of realism is injected into the deployment process.

### End users

In 1999, Adams and Sasse wrote a seminal paper titled “Users are not the Enemy”. This paper played a crucial role in introducing the need to acknowledge the role of humans in cyber security to industry and academia. These authors are psychologists. Even so, both software engineers and cyber security researchers benefited from collaborations with psychologists since Adams and Sasse highlighted the need to accommodate the human in cybersecurity.

**Fig. 1:**
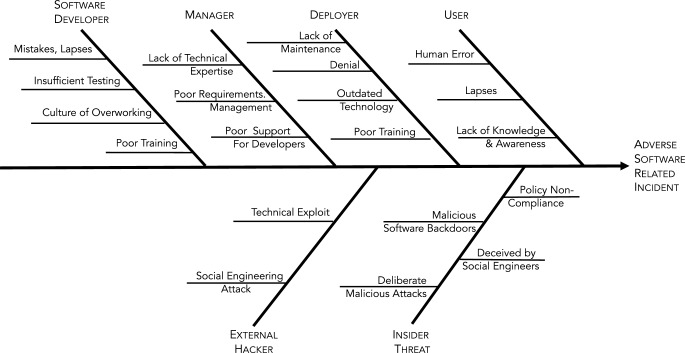
Summary of Human Factors affecting Software Engineering (Upper row is reflects unintentional, while the lower is intentionally malicious) Zusammenfassung menschlicher Faktoren, die sich auf die Softwareentwicklung auswirken (obere Reihe spiegelt unbeabsichtigt wider, während die untere absichtlich böswillig ist)

Even so, many still refer to the human as the “weakest link” two decades later. Zimmermann and Renaud (2019) demonstrate that this attitude is not merely anecdotal, but is entrenched—almost an unquestioned stereotype. In fact, while humans do indeed make errors, and sometimes behave insecurely, Fig. 1 shows the wide range of sources of issues that can cause an adverse software-related incident.

Humans sometimes make errors that subvert security measures. Moustafa et al. (2021) enumerate a number of these, which include poor password management, oversharing, being deceived by social engineers not updating software on their devices. Much of this is due to a lack of awareness, but also because users do not have the tools that could help to eliminate these behaviours, most of which are purely coping strategies. For example, password managers alleviate password memorial loads, and consequently improve password strength. However, they have not really diffused through the population (Alkaldi and Renaud 2022) and few organisations provide these to their employees. There are also tools to help people to spot Phishing messages more effectively, but these, too, are not widely used by organisations. A focus on the supporting rather than blaming is likely to reduce insecure behaviours (Renaud et al. 2021b).

An overview of the literature dealing with the impact of human nature on the secure software engineering process is provided in Table 1 in the Appendix, and in Fig. 1.

## Implications for practice & research

**Fig. 2:**
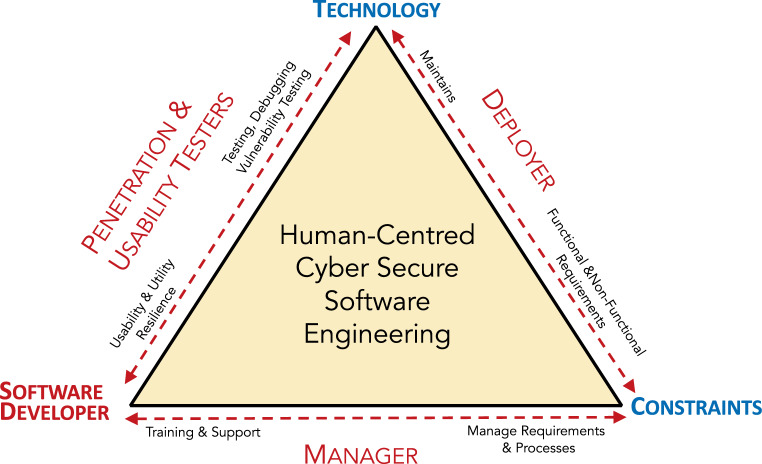
Human-Centred Cyber Secure Software Engineering Triangle Menschenzentrierte Cybersicherheit Software-Engineering-Dreieck

Fig. 2 depicts all the roles and considerations that come into play in the human-centred cyber secure software engineering process.

Now, we address the second research question: “*How can the impact of human fallibility be ameliorated during the secure software engineering process?”*

### Practical implications

There are some practical implications if we are to engender the production of secure software systems:

1. Managers should:
  - ensure that software engineers are supported in their need to develop mastery as much as possible, since this appears to be the need that this discipline is particularly suited to satisfy.
  - Software engineers ought to be trained not merely to secure the system against known threats, but also to anticipate vulnerabilities that could be exploited, and then to actively prevent these.
  - Support the engineers in crafting systems that accommodate human tendencies to make mistakes. Given them time to allocate to usability and security-related activities.
  - Do not pressure developer to the extent that they are unable accommodate human fallibility and malice.
2. Deployers should:
  - test software regularly to ensure that newly emergent vulnerabilities are addressed,
  - be realistic in accepting that software is seldom perfect and considering this possibility before blaming end users when anomalous outcomes occur.

### Research implications

There are many avenues for research in this area. Three urgently need attention:

1. Software developers need to be supported more effectively in developing secure software, as highlighted in the research (Sect. 3), and in order to satisfy their personal mastery needs. This is a challenging area because the goalposts change all the time as new exploits emerge, and developers are under pressure to complete the. product so that it can be deployed as soon as possible. This reality should be built into the training programmes and also into their delivery milestones. Developing tools to support managers would be helpful. Both training and tools would be fruitful avenues for future research.
2. There is a naivety amongst the general public about the complexity of software development and the challenges of keeping software running correctly and without being compromised. In order to inject a measure of realism into expectations of software, there is a need to develop a public relations campaign and some short courses for those in other walks of life to help them to develop an appreciation of the complexities of software engineering. This requires a rigorous research endeavour from communications researchers.
3. There is a need to change the industry mindset away from “user as problem”. This has occurred in other more mature fields such as safety (Dekker 2018) and medicine (Berry 2022). Those doing research in the software engineering domain have a role to play in adapting the lessons from these fields for the software engineering discipline.

## Conclusions

The software engineering field is huge and enjoys attention from many researchers. The cyber security field, too, is extensive. Here, I have sought to provide an overview of the human-related aspects of the secure software engineering domain. The biggest lessons I would like readers to take away are the following: (1) wherever humans are involved in any endeavour, it benefits us to learn from those who understand human nature best: psychologists. We should not expect perfect performance from any human, and accommodate that in our expectations of human stakeholders in the software engineering domain. (2) No one benefits if a profession is shrouded in mystery. During the pandemic, everyone became familiar with terms such as coronavirus, spike protein and cytokine storm. This new familiarity helped public health officials to communicate with the general public. The software engineering profession should start helping the public to understand the complexity of their profession too. If they do, everyone will benefit.

## Appendix

**Table 1:** Secure Software Development Issues Probleme bei der sicheren Softwareentwicklung

	Plan	Analyse	Design	Develop	Test	Deploy	Maintain
Programmers	Not recruiting enough coders (Lehtinen et al. 2014)	Not understanding deployment context & constraints (Dyson and Longshaw 2004)	Staff Inexperience (Shahzad et al. 2011) Not understanding how the software interacts with existing systems (Bosch 2010)	Lack of Cooperation (Lehtinen et al. 2014) Insecure coding (Du and Mathur 1998) Tradeoffs between reuse and redevelopment (Basili and Perricone 1984)	Inadequate test cases (Meenakshi et al. 2014) Not understanding users (Borenstein 1991)	No audit trail or error logging (Ko et al. 2007)	–
Project Managers	Flawed Planning (Pinto 2013) Underestimating the amount of time needed for planning (Brooks 1975)	–	–	Weak Task Backlog (Lehtinen et al. 2014) Poor monitoring (Brooks 1975) Underestimating effort (Gray et al. 1999)	Lack of Resources (Lehtinen et al. 2014) Shortening testing time (Westland 2002)	Optimism Bias & Over-promising (Pinto 2013)	–
Deployer	–	Immature Requirements (Shahzad et al. 2011)	Creeping user requirements (Harker et al. 1993)	–	Pressure for Deployment (Pinto 2013; Jones 1993)	Poor Training (Wallis 2021)	Blaming users for errors (Clark 2021)
Security	Not integrating security throughout the life cycle (Khan et al. 2021; Mouratidis et al. 2005)	Incomplete Security Requirements (Sultan et al. 2008)	Not designing for security (Sultan et al. 2008)	Implementation Errors (Sultan et al. 2008) Trying to bolt security on after development (Pearlson and Huang 2022)	Inadequate Testing (Sultan et al. 2008)	Architectural security flaws (Abeyrathna et al. 2020)	Not staying on top of new threats (Bernsmed et al. 2022; Devanbu and Stubblebine 2000)

## Footnotes

1 https://alec.org/article/colonial-pipeline-and-darkside-cyber-attacks-have-real-world-consequences.

2 https://slidetodoc.com/debugging-is-twice-as-hard-as-writing-the/.

3 https://www.wsj.com/articles/roundtable-the-cio-in-an-age-of-disruption-11649066400?mod=djemCybersecruityPro&tpl=cy.

4 https://owasp.org/www-project-top-ten/.
